# Molecules Produced by Probiotics and Intestinal Microorganisms with Immunomodulatory Activity

**DOI:** 10.3390/nu12020391

**Published:** 2020-02-01

**Authors:** Susana Delgado, Borja Sánchez, Abelardo Margolles, Patricia Ruas-Madiedo, Lorena Ruiz

**Affiliations:** 1 Department of Microbiology and Biochemistry of Dairy Products, Dairy Research Institute of Asturias (IPLA)-Spanish National Research Council (CSIC), Villaviciosa, 33300 Asturias, Spain; sdelgado@ipla.csic.es (S.D.); borja.sanchez@csic.es (B.S.); amargolles@ipla.csic.es (A.M.); ruas-madiedo@ipla.csic.es (P.R.-M.); 2 Instituto de Investigación Sanitaria del Principado de Asturias (ISPA), Oviedo, 33011 Asturias, Spain

**Keywords:** microbiota, microbiome, probiotics, immunomodulation, molecular effectors

## Abstract

Probiotics are live microorganisms that, when administered in adequate amounts, confer a health benefit on the host. The probiotic microorganisms most commonly used in the food and pharmacy industry belong to *Lactobacillus* and *Bifidobacterium*, and several strains of these genera have demonstrated beneficial attributes. In addition, some other intestinal bacteria inhabiting the human microbiota, such as *Faecalibacterium prausnitzii* and *Akkermansia muciniphila*, have recently been discovered and are able to display health-promoting effects in animal and human trials. The beneficial properties of probiotics have been known for a long time, although little is known about the molecular mechanisms and the molecules responsible for their effects. However, in recent years, advances in microbiome studies, and the use of novel analytical and molecular techniques have allowed a deeper insight into their effects at the molecular level. This review summarizes the current knowledge of some of the molecules of probiotics and other intestinal commensal bacteria responsible for their immunomodulatory effect, focusing on those with more solid scientific evidence.

## 1. Introduction

Some health-promoting effects of gut commensal, beneficial and probiotic microorganisms can be mediated through soluble factors (products or metabolic byproducts), secreted by live bacteria, or released after bacterial lysis. Precise identification of these biologically active compounds will enable a better formulation of microbiome-based biotherapeutics, and open up the possibility of administering purified biologically active fractions rather than whole live cells [1]. Besides, numerous other beneficial effects of gut microorganisms are mediated through their key role in the modification of dietary precursors within the gut ecosystem. These modifications can lead to the production of soluble bioactive fractions such as short chain fatty acids (SCFA), including butyrate, acetate and propionate, which have been correlated with health deterioration in the elderly [2,3]. Also, it has been suggested that some amino acid derivatives, such as indole, display lower fecal concentration levels in patients suffering from ulcerative colitis [4]. Indeed, using microbial bioactive compounds rather than live microorganisms could be particularly important when dealing with vulnerable immunocompromised or immunodeficient populations. Overall, these cellular or metabolic fractions can have immunomodulatory, antihypertensive, anti-proliferative and anti-oxidant activities, representing the mechanism of action for the health-promoting traits attributed to some gut commensals. 

Traditionally, the concept of health promotion through gut ecosystem modulation has relied either on the administration of live cells of gut commensals with probiotic attributes, usually belonging to a narrow range of microbial species, or in the administration of prebiotic substrates capable of sustaining such populations within the gut ecosystem [5]. However, recent evidence is leading to the inevitable evolution of the strategies available to modulate the gut ecosystem. The identification of the soluble mediators of the health-promoting effects of gut microbes implies that improvement in the well-functioning gut ecosystem and host well-being might be independent of probiotic cells viability, thus facilitating novel opportunities for gut microbiota-mediated health promotion [1]. This might be particularly important in the framework of recent investigations, which are pointing towards the existence of a much wider range of health-promoting commensal microorganisms within the healthy gut ecosystem than previously anticipated [6]. As such, current trends in the field are focusing their attention on the potential health-promoting traits of commensal bacterial species which do not fall into the traditional probiotic groups, *Bifidobacterium* and *Lactobacillus*, but into well-adapted gut microorganisms such as *Faecalibacterium*, *Akkermansia*, *Ruminococcus* and other *Lachnospiraceae* members, whose extreme oxygen sensitivity and tight adaptation to the gut ecosystem severely hampers their preparation as viable (food) supplements. Those microorganisms could be referred to as next-generation probiotics [6]. Identification of biologically active fractions from such microorganisms will undoubtedly open novel possibilities to exploit their health-promoting attributes, avoiding the technological and regulatory hassle of converting them into viable whole-cell supplements. However, despite the great advances that precise delineation of the biologically active fractions of gut commensals will have for the design of microbiota-based health promotion strategies, only a few cellular components or metabolites from gut commensals have been unequivocally associated with health promotion mechanisms up to date.

The present review summarizes current evidence on cellular and metabolic soluble fractions of gut commensals for which a health-promoting effect, mainly immunomodulation, has been attributed, including their metabolic action on selected dietary bioactive ingredients. Besides, it will discuss the possible exploitation of this knowledge to design novel gut microbiome-based biotherapeutics and food supplements capable of ameliorating an increasingly large range of health conditions. A summary of the immunomodulatory molecules and compounds discussed in this review is presented in Table 1.

## 2. Proteinaceous Molecules with Immunomodulatory Activities

Extracellular or surface-associated proteinaceous molecules from commensal gut microorganisms exert crucial functions in their interaction with the host, and represent important microbe-associated molecular patterns (MAMPs), leading to the activation of specific signalization pathways upon pattern recognition receptors (PRRs) recognition. For this reason, some of them have been identified as the molecular effectors of some health-promoting attributes mediated by probiotic and commensal microorganisms [7,8]. Indeed, specific extracellular and secreted proteins from commensal microorganisms are the primary target of intestinal IgA, whose main role is to monitor the commensal bacterial populations within the gut. In this context, up to six different extracellular proteins from *Bifidobacterium longum*, *Bifidobacterium bifidum* and *Bifidobacterium animalis* strains were recognized by pooled sera from healthy volunteers or inflammatory bowel disease (IBD) patients [9]. Also, levels of IgA antibodies developed against a cell-wall hydrolase from *Lactobacillus rhamnosus* GG were significantly higher in the IBD group, indicating that IBD patients appeared to have different immune response to food bacteria [9]. In the same way, remarkably, surface immunoreactive proteins were identified in 4 lactobacilli strains, including the presence of several moonlighting proteins, represented by proteins lacking any known secretory motif, but known to be located in the microbial surface [10].

In relation to the specific proteins from commensal microorganisms which might trigger immune effects in the host, pili-structures are common mediators of the immunomodulation exerted by various commensal species, including the classical lactobacilli or bifidobacterial groups. For instance, *Lactococcus lactis* heterologously expressing pili-proteins from *B. bifidum* associated with reduced IL10 and increased tumor necrosis factor α (TNFα) in an in vivo mice model [11]; and co-administration of flagellin and *Lactobacillus casei* strain Shirota, enhanced IL12 production [12]. Besides, two secreted proteins from lactobacilli, p75 and p40, have been found capable of inhibiting cell apoptosis induced by proinflammatory states [13]. A novel secreted protein from *L. rhamnosus* GG, HM0539, has been deemed as being responsible for the protective effects exhibited by the corresponding producing strain on the intestinal barrier, enhancing intestinal mucin expression and preventing lipopolysaccharide (LPS) or TNFα-induced intestinal barrier injury [14]. 

Recent investigations have revealed that, in addition to classical probiotic species, other gut commensal microorganisms frequently dominant in healthy populations and depleted in several disease population groups, can also interact with the host immune system in a beneficial way, and might thus represent promising next generation health-promoting microorganisms. In a few cases, proteinaceous determinants of their health-promoting attributes have also been identified. These include both homologues to proteins present in lactobacilli and bifidobacterial species, but also other specific novel proteins. For instance, pili-structures from *Akkermansia muciniphila*, a microorganism which has been attributed a critical role in the amelioration of metabolic syndrome and type-2 diabetes, have also been identified as key mediators of the immune and gut barrier function [15]. Concerning other specific mediators of the effects exerted by other beneficial gut commensals, seven peptides secreted by *Faecalibacterium prausnitzii*, all belonging to a protein called the microbial anti-inflammatory molecule (MAM), showed anti-inflammatory properties both in vitro and in in vivo colitis models, inhibiting nuclear factor (NF)-kB pathways and reducing T-helper (Th)1 and Th2 responses [16]. Besides, immunoglobulin binding super antigenic proteins, IbpA and IbpB, have also been identified in *Ruminococcus gnavus*, a member of the taxon that defines one of the enterotypes that describes the gut microbiota inter-individual variability [17]. IpbA and IpbB expression have been associated with high IgA coating of the corresponding strain in vivo, as well as higher serum IgA levels [18]. Remarkably, about 40% of the analyzed gut metagenomes from healthy individuals from the United States and China presented these superantigenic proteins, yet their specific contribution to health and disease states remains to be elucidated.

Recent investigations have also delineated specific short peptide sequences, encrypted in larger surface-associated or extracellular proteins from commensal microorganisms, responsible for the observed immunomodulatory effects exerted by the producing strains. As an example, the STp peptide, a serine and threonine rich peptide included in one of the main extracellular proteins from *Lactobacillus plantarum*, was the first to be described as inducing an anti-inflammatory response in dendritic cells from IBD patients [19,20]. More recently, the development of proteomic databases of the gut microbiome has enabled the prediction of immunomodulatory potential of peptides encrypted in the gut commensals metaproteome [21]. Such an approach has led to the identification and validation of the immunomodulatory potential of two encrypted peptides, FR-16 and LR-17, included in proteins from *B. longum* DJO10A and *Bacteroides fragilis* YCH46, which exhibited a capacity to differentiate Th17 and Th22 differentiation pathways using immune cells models [7]. These works evidence that precise identification of specific proteins and/or encrypted peptides from gut commensals mediating immune responses in the host, will lead to the design of novel gut microbiota-based biotherapeutics.

## 3. Exopolysaccharides and Immunomodulation

The most external layer covering the surface of many bacteria is constituted by repeating units of monosaccharides, building a polymeric matrix of exopolysaccharides (EPSs) which can be loosely attached forming a slime structure, or be covalently linked as a capsule. Certain EPSs with specific characteristics, such as the zwitterionic (having both positive and negative charges) polysaccharides produced by *Bac. fragilis*, play a crucial role in the maintenance of the immune homeostasis at the intestinal level [22]. In fact, the presence of both charges is of pivotal relevance for activation of the immune response since this capability is lost when the polymers are modified to change the charge of the molecule. In *Lactobacillus* spp., the polymers having a single type of monosaccharide are known as homopolysaccharides (α-glucans, β-glucans or β-fructans). In this genus, as well as in *Bifidobacterium* spp., heteropolysaccharides, which are those having different monosaccharides (typically D-glucose, D-galactose and L-rhamnose), are also synthesized [23]. Both EPS types constitute a protective cover for the producing bacterium, but also act as an MAMP that will be recognized by different PRRs, thus inducing different responses in the host [24]. Little is known about the PRRs involved in the recognition of EPS synthesized by probiotics. It has been shown that specific β-glucans could act as ligands for certain C-type lectins (such as Dectin 1) located in the intestinal epithelium [25]; additionally, specific heteropolymers synthesized by bifidobacteria or lactobacilli can be recognized by TLR (toll like receptor)-4 [24,26,27]. 

Some of the health benefits exerted by probiotics have been attributed to the presence of EPS structures in the envelope surrounding the producing bacteria [23,28]. Immunomodulatory potential has been suggested for polymers synthesized by lactic acid bacteria and bifidobacteria having specific characteristics, such as high molar mass (more than 10^6^ Da), or the presence of a negative charge in the molecule [29,30]; however, the mechanism by which these specific bacterial EPSs trigger immune response remains to be elucidated. More recently, it has also been indicated that the presence of galactose in the EPSs synthesized by *Lactobacillus reuteri* strains enhanced their anti-inflammatory effects on macrophages, although the scarce number of polymers analyzed limited a conclusive statement [31]. Additionally, most of the evidence on the immunomodulation capacity of probiotics EPS was based on in vitro observations, mainly using cultures of peripheral mononuclear cells (PBMCs), dendritic cells, or macrophage cell lines, after checking the pattern of cytokines released [32]. Nonetheless, in recent years, the protective effect of EPS-producing probiotics on immune compromised states has also been studied in vivo using animal experimental models. *F. prausnitzii*, one of the most abundant members of Firmicutes phylum in the colon of healthy individuals, is underrepresented in patients with IBD; therefore, it has been proposed as an anti-inflammatory next-generation probiotic. The EPS matrix produced by this species seems to be directly correlated with its immunomodulation capability as was demonstrated after an intra-rectal administration of the purified EPS in a model of acute ulcerative colitis (DSS-induced) BALB/c mice. The in vitro analyses with immune cells suggested that the anti-inflammatory potential of the EPS synthesized by *F. prausnitzii* HTF-F was mediated by production of IL-12 and IL-10 in antigen presenting cells in a TLR-2-dependent manner [33]. In a similar way, the oral administration of *B. animalis* subsp. *lactis* Balat_1410^S89L^ to DSS-induced colitis C57BL/6J mice reduced the damage (disease activity index) caused by this chemical agent. This strain produced a “ropy” EPS, having a high molar mass (about 10^6^ Da) with a high content of rhamnose (more than 50%), and formed a long filament when the colony growing on the surface of agar-culture was touched with an inoculation loop. The comparison of the ropy strain with its non-ropy isogenic counterpart, showed that the attenuation of the damage caused by DSS could be related with the capability of the first to induce an increase in the T regulatory (Treg) cell number of mesenteric lymphoid nodes, which could lead to a reduction in the inflammatory state at the mucosal level [34]. In fact, previous ex vivo experiments carried out with these two isogenic *B. animalis* subsp. *lactis* strains showed the high anti-inflammatory potential of the ropy EPS-producing strain. This ropy Balat_1410^S89L^ strain favored a significant increase of IL-10 by human PBMCs after co-cultivation with an UV-inactivated bifidobacterial suspension; similarly, co-incubation with colonic biopsy specimens significantly reduced the production of TNFα by the tissue [35]. Thus, *B. animalis* subsp. *lactis* strains having a ropy phenotype, denoting the production of EPS with specific traits, could be proposed as probiotic candidates to attenuate intestinal inflammatory episodes. More recently, similar in vivo works corroborate these findings with different EPS-producing bifidobacteria. The EPS synthesized by *Bifidobacterium adolescentis* IF1-03 was able to modulate the macrophage-regulated Treg/Th17 axis and, therefore, protect DSS-colitis mice [36]; and the EPS-producing *B. longum* subsp. *longum* YS108R alleviated the DSS-induced colitis by modulating inflammatory cytokines, reinforcing the mucosal barrier and also reverting the microbial dysbiosis caused by this chemical agent [37]. 

The results briefly summarized above, demonstrating the immune modulating capability of EPS synthesis by Gram-positive probiotics, show a promising way of intervention to attenuate intestinal inflammatory states. Additionally, the viability of the producing bacterium is not required to exert a beneficial effect and the EPS layer covering the bacterial surface is resistant to the harsh conditions of the gastrointestinal tract, thus allowing it to arrive intact to the colon [38]. Therefore, the use of EPS-producing strains from difficult-to-handle bacteria, such as, for example, those that lost viability in the presence of oxygen, could be a solution to overcome the technological challenge that currently supposes the production of some next generation probiotics. 

## 4. Other Cell-Wall Components

In addition to EPS and proteinaceous components that have already been discussed in previous sections, the biological layers surrounding the bacterial cell membrane are a reservoir of other macromolecules with immunomodulatory activity. Among them, the cell wall of Gram-positive bacteria has been the most studied and numerous articles show that cell wall fractions display different bioactive functions. For example, exposure of dendritic cells to cell wall extracts from *B. bifidum* trigger Treg differentiation [39], and it has been shown that the immune stimulatory properties of *L. plantarum* in macrophage cells are largely due to cell wall crude fractions [40]. Perhaps the most studied immune-active molecules contained within the cell wall are teichoic and lipoteichoic acids (TAs and LTAs, respectively). In this regard, very little information is available about these molecules in *Bifidobacterium* and few papers have described the role of bifidobacterial LTAs in immune responses [41]. On the contrary, some pioneering works show that the TA and LTA of *Lactobacillus* species can have a potent effect on the immune system, and their role has been elucidated using in vitro and animal models. Grangette et al. [42] evaluated the role of TAs in the interaction between *L. plantarum* NCIMB8826 and the immune system by analyzing the anti-inflammatory properties of a mutant affected in the TA biosynthesis. The deficient mutant, having a lower content of alanine in its TA, was able to increase IL-10 production and dramatically reduce secretion of pro-inflammatory cytokines by PBMCs and monocytes, as compared with the wild type strain. Furthermore, the mutant was more protective in a murine colitis model. The results indicated that LTA of *L. plantarum* can modulate pro-inflammatory or anti-inflammatory responses. Subsequent studies showed other immunomodulatory features of *L. plantarum* LTA, such as the down-regulation of *Shigella flexneri* peptidoglycan-induced inflammation [43], or the suppression of LPS-mediated inflammation [44], among other effects. LTA function has also been studied in *L. rhamnosus* GG (LGG), one of the most studied probiotics [45]. Mice treated with an LTA deficient mutant of LGG showed an improvement of colitic parameters compared to LGG wild-type-treated mice; an effect which was likely mediated by LTA-TLR2 interactions [46]. Also, Claes and coworkers [47] showed that LTA of LGG is an MAMP with pro-inflammatory activities via TLR2/6 interaction. Furthermore, a recent work demonstrated that LTA of LGG primes the epithelial stem cell niche to protect epithelial stem cells from radiotherapy, by promoting an adaptive immune signaling cascade [48]. The beneficial effects of LTA have also been demonstrated in the species *Lactobacillus paracasei*. Its LTA enhances mucin expression by modulating the TLR-2 pathway, and reduces leaky gut and inflammation [49]. Overall, current evidence indicates that TAs and LTAs from probiotic bacteria interact with the host to play important physiological roles as potent immune modulators [50].

## 5. Microbial Metabolites with Immune Function 

Intestinal microorganisms regulate the host immune system, in part, by producing metabolites acting via host receptors and other target molecules. Immune cells express receptors specific for metabolites, such as aryl hydrocarbon receptor precursor (AhR), pregnane X receptor (PXR) and farnesoid X receptor (FXR) among others, or less specific receptors like cell surface G-protein-coupled receptors (GPCRs) as an example [51]. Microbial metabolites can have a bidirectional function to promote both tolerance and immunity. Some of the microbial metabolites with immunomodulatory functions are discussed next.

### 5.1. -Short-Chain Fatty Acids 

The microbial metabolites better exemplifying this are the short-chain fatty acids (SCFAs) acetate (C2), propionate (C3) and butyrate (C4), produced as the result of bacterial carbohydrate fermentation in the colon [52]. These SCFAs act on leukocytes and endothelial cells through at least two mechanisms: activation of GPCRs receptors and inhibition of histone deacetylase (HDAC) [53]; however, the production of these SCFAs differs greatly among the intestinal members. Acetate is generated by many genera of intestinal microorganisms, including *Bifidobacterium* spp. Acetate released in the gut is used as substrates for other microbial gut fermenters, mainly butyrate and propionate producers belonging to the clostridial cluster IV and XIVa [54]. The production of these two major SCFA metabolites has been shown to have anti-inflammatory effects and promote and regulate the colonic Treg cells pool [55]. In particular, butyrate, as an epigenetic regulator of gene expression, acts on both DNA methylation and histone hyperacetylation [56]. By inhibiting histone deacetylation, butyrate acts in the differentiation of Treg cells, increasing the expression of the Treg marker *Foxp3*^+^ [57]. Also, acetate increases the acetylation of the Foxp3 promotor. Among the SCFAs, butyrate is the most potent HDAC inhibitor and acetate is the least potent [58]. Propionate, which is primarily produced by Bacteroidetes and some Firmicutes members of the intestinal microbiota mainly via the succinate metabolic pathway, has also been described as educating dendritic cells to achieve high phagocytic capacity and affect Th2 cell responses [59].

As previously mentioned, SCFAs also activate several GPCRs and, probably, the best-characterized are GPR43, GPR41 and GPR109A. GPR43 and GPR109A appear to be important for gut homeostasis, and both are expressed by intestinal epithelial cells, but not by T- and B- immune cells [60]. Some other immune populations, such as dendritic cells and inflammatory leukocytes (such as neutrophils and macrophages), express these GPR43 and GPR109A receptors at variable levels [58]. However, the study performed by Trompette and colleagues [59] proposes the effects of propionate on allergic inflammation to be dependent on GPR41, but not the GPR43 receptor. This is one of the reports showing the effect of increased levels of SCFAs and activity of their receptors GPCRs on enhancing oral tolerance and protecting against allergy [59,61]. The GPR109A receptor has recently emerged as a major regulator of gut homeostasis; this binds butyrate, but also the tryptophan metabolite nicotinic acid that will be described in the following section.

In addition to the interaction with different receptors, indirect evidence suggests that SCFAs might also promote the secretion of IgA by B immune cells [62]. Besides, the ability of SCFAs to inhibit the NF-κB is already known and SCFAs have been reported to reduce production of inflammatory chemokines and cytokines such as TNFα, IL-6, and interferon-γ (IFN-γ) [53]. The anti-inflammatory effects of SCFAs on chemotaxis and leukocyte recruitment have been documented [63].

Unlike what happens with the three main intestinal SCFAs acetate, propionate and butyrate, little is known about the immunomodulatory effects of other SCFAs like the branched chain fatty acids (BCFAs) isobutyric and isovaleric acids, which are more related to microbial catabolism of proteins [52]. However, they are also reported in literature as HDAC inhibitors; therefore, their function in regulating host cells is expected to be similar to that of butyrate [51].

### 5.2. -Compounds Derived from Protein Degradation 

The gut microbiota can determine to what extent dietary proteins are converted into other active metabolites, such as BCFAs, or different nitrogen containing compounds which could play a major role in the prevention of inflammatory diseases and are highlighted for their interaction with the immune system [58]. 

Other microbial metabolites from the breakdown of proteins are indolic compounds, mainly derived from aromatic amino acids like tryptophan. Tryptophan is degraded to skatole (3-methylindole) and other indoles by microbial degradation in the intestine with diverse gut microorganisms, such as lactobacilli [58]. The bioactive indole-3-aldehyde, indole-3-propionato and indole-3-acetic acid are products of the bacterial metabolism of tryptophan that affect the intestinal barrier integrity and immune cells activity through the activation of PXR and AhR receptors [64,65]. *L. reuteri* is one of the tryptophanase-positive bacteria that generate these indole metabolites and can stimulate AhR activity suppressing pro-inflammatory activities [65,66]. Other gut members such as *Peptostreptococcus* produce 3-indoleacrylic acid (IA) from tryptophan which promotes epithelial barrier function and mitigates inflammatory responses [67]. In fact, the metabolism of tryptophan has been related to allergy through its degradation via the immune-regulatory enzyme indoleamine 2,3-dioxygenase-1 (IDO-1), which is activated by the IFN-γ [68]. IFN-γ is a strong inducer of IDO-1, which degrades this essential amino acid as part of an immunoregulatory strategy to avoid over-activation of the immune system [68]. IDO activity is linked to suppression of T cell responses, promotion of Treg cells and immune tolerance [69]. Other reports exist indicating that D-tryptophan from probiotic bacteria can decrease Th2 response in the gut and lung, influencing allergic reactions [70,71]. Moreover, tryptophan is degraded endogenously via this IDO enzyme to kynurenin (an AhR agonist) which, after binding to Ahr receptors, promotes the production of important mediators for gut homeostasis [58]. Other tryptophan metabolites, including kynurenic acid and niacin, also target metabolite-sensing GPCRs, such as GPR35 and GPR109A. From these, GPR109A binds the SCFA butyrate but also the tryptophan metabolite nicotinic acid which is known to have anti-inflammatory properties [58]. It is necessary to clarify that all these kyneurines are endogenous (host) tryptophan metabolites different from the bacterial tryptophan metabolites (indole metabolites) [72]. 

**Table 1:** Immunomodulatory molecules and compounds discussed in this review.

Species	Molecule	Effect	Reference
**Peptides and proteins**			
*Bifidobacterium* sp.	Pili	↑TNFα, ↓IL10	[11]
Several species	Flagellin	↑IL12	[12]
*Lactobacillus* sp.	p75 and p40	↓Cell apoptosis	[13]
*Lactobacillus rhamnosus*	HM0539	↓LPS- or TNFα-mediated barrier injury	[14]
*Akkermansia muciniphila*	Pili-structures	Amelioration of metabolic syndrome and type-2 diabetes	[15]
*Faecalibacterium prautsnitzii*	MAM	↓NF-kB pathways, ↓Th1 and Th2 responses	[16]
*Ruminococcus gnavus*	IbpA and IpB	Targets for IgA coating	[18]
*Lactobacillus plantarum*	STp	↑IL10, ↓IL12	[19,20]
*Bifidobacterium longum*	FR-16	↑Th17, Th22 responses	[7]
*Bacteroides fragilis*	LR-17	↑Th17, Th22 responses	[7]
**Exopolysaccharides**			
*Bacterioides fragilis*	Zwitterionic	Homeostatic molecule	[22]
Several species	β-glucans	C-type lectin ligands	[25]
Bifidobacteria/Lactobacilli	Heteropolymers	TLR-4 ligands	[24,27]
Bifidobacteria/Lactobacilli	High Molar EPS	Immunomodulatory	[29]
*Lactobacillus reuteri*	EPS	Anti-inflammatory	[31]
*Faecalibacterium prausnitzii*	EPS	↑IL-12 and IL-10; TLR-2-mediated	[33]
*Bifidobacterium animalis* subsp. *lactis*	Ropy EPS	↑IL10	[35]
*Bifidobacterium animalis* subsp. *lactis*	Ropy EPS	↑Treg response	[34]
*Bifidobacterium adolescentis*	EPS	Treg/Th17 response modulation	[36]
*Bifidobacterium longum*	EPS	Inflammatory cytokine modulation, mucosal barrier reinforcement	[37]
**Other cell wall components**			
*Bifidobacterium bifidum*	Cell wall extract	Treg differentiation	[39]
*Lactobacillus plantarum*	Cell wall extract	Immunostimulatory	[40]
*Lactobacillus plantarum*	Teichoic acids	IL10 production modulation	[42]
*Lactobacillus plantarum*	Lipoteichoic acids	Suppression of LPS-mediated inflammation	[44]
*Lactobacillus rhamnosus*	Lipoteichoic acids	Proinflammatory, TLR-2/6 ligand	[46,47]
*Lactobacillus rhamnosus*	Lipoteichoic acids	Radiotherapy protection	[48]
*Lactobacillus paracasei*	Lipoteichoic acids	↓Leaky gut and inflammation	[49]
**Microbial metabolites**			
Several species	Short-chain fatty acids	Activation of GPCRs ↓histone deacetylase	[53]
Several species	Short-chain fatty acids	NF-κB inhibition, ↑IgA secretion, ↓pro-inflammatory cytokines, ↑leukocyte recruitment	[63]
Several species	Propionate, butyrate	Anti-inflammatory, ↑Treg response	[55]
Several species	Butyrate	↓histone deacetylase, ↑ Treg	[57]
Several species	Propionate	Affects Th2 response	[59]
Several species	Branched-chain fatty acids	Inhibition of histone deacetylase	[51]
Several species	Indole-3-aldehyde, indole-3-propionate and indole-3-acetic acid	↑Barrier integrity and immune cell function	[64,65]
*Peptostreptococcus sp.*	3-indoleacrylic acid	↑Epithelial barrier and immune cell function	[67]
Several species	D-tryptophan	↓Th2 response	[70,71]
Several species	Kynurenic acid, niacin, nicotinic acid	Gut homeostasis regulators, anti-inflammatory	[58]
Several species	Glutamine, histidine and glycine- derived metabolites	Influence gut homeostasis and immune cell function	[70]

## 6. Future Perspectives

Immunomodulation is one of the most studied characteristics of probiotic microorganisms. The ability to stimulate our immune function has especially been studied in lactobacilli and bifidobacteria, where some molecules responsible for beneficial effects (such as surface proteins and EPS) have been characterized. However, currently, the scientific community has a variety of novel methodologies that allow the study of the probiotic-mediated immunomodulation from novel perspectives. The new techniques used in microbiomic studies [73], genomic editing such as CRISPR-Cas [74], and cell and molecular biology [75], facilitate much more efficiently the unraveling of the role of the molecules and pathways responsible for the mechanisms of action of probiotics. This will lead to the selection of probiotics with activities aimed at personalized treatments, based on solid knowledge of their effector molecules.
